# Role of L-carnitine in female infertility

**DOI:** 10.1186/s12958-018-0323-4

**Published:** 2018-01-26

**Authors:** Ashok Agarwal, Pallav Sengupta, Damayanthi Durairajanayagam

**Affiliations:** 1 American Center for Reproductive Medicine, Cleveland Clinic, Glickman Urological and Kidney Institute, Mail Code X-11, 10681 Carnegie Avenue, Cleveland, OH 44195 USA; 2 Department of Physiology, Faculty of Medicine, MAHSA University, Jalan SP2, Bandar Saujana Putra, 42610 Jenjarum, Selangor Malaysia; 3 Faculty of Medicine, Universiti Teknologi MARA, Sungai Buloh Campus, Jalan Hospital, 47000 Sungai Buloh, Selangor Malaysia

**Keywords:** Assisted reproductive technology, Acetyl-L-carnitine, Antioxidants, Female fertility, In vitro fertilization, L-carnitine, Oocyte quality

## Abstract

**Background:**

L-carnitine (LC), and its acetylated form, acetyl L-carnitine (ALC), have immense functional capabilities to regulate the oxidative and metabolic status of the female reproductive system. The vulnerability of this system to free radicals demand for advanced strategies to combat them. For this purpose, the ‘quasi vitamins’ LC and ALC can be used either individually, or in combination with each other or with other antioxidants.

**Main body:**

This review (a) summarizes the effects of carnitines on female fertility along with the findings from various in vivo and in vitro studies involving human, animal and assisted reproductive technology, and (b) proposes their mechanism of actions in improving female fertility through their integrated actions on reducing cellular stress, maintaining hormonal balance and enhancing energy production. They reportedly aid β-oxidation in oocytes, maintain its cell membrane stability by acetylation of phospholipids and amphiphilic actions, prevent free radical-induced DNA damage and also stabilize acetyl Co-A/Co-A ratio for adequate acetyl storage as energy supply to maintain the robustness of reproductive cells.

**Conclusion:**

While both LC and ALC have their applications in improving female fertility, ALC is preferred for its better antioxidant properties and LC for amelioration of energy supply to the cells. These beneficial effects show great promise in its application as a treatment option for women facing infertility disorders.

## Background

The revolution of modern day reproductive biology research and assisted reproductive technology (ART) are promising to provide a more exhaustive description of the effects of metabolic supplementation on infertility. To date, thousands of supplements have been proposed to mitigate the outcomes of reproductive disorders, but most of them have ended up having several side-effects on other systems [1]. In order to combat this, reproductive biologists are currently searching for some supplements, or combination of supplements to fight against the problems of infertility. In this aspect, mitochondrial metabolism of both the male and female germ cells exert a crucial role [2]. Mitochondrial nutrient supplementation is also reported to be effective in many cases [3].

Levocarnitine or L-carnitine (LC) is a biologically active stereoisomer of 3-carboxy-2-hydroxy-N, N, N-trimethyl-1-propanaminium. It exists as a highly polar, small zwitterion (an ion that while electrically neutral, carries both a positive and a negative electrical charge in different parts of the molecule, as seen in certain amino acids and protein molecules) [4]. LC is reported to have benefits in the management of infertility [5, 6]. The impact of LC on male infertility is now well documented. It has been reported to be concerned with epididymal maturation of spermatozoa [7]. LC serves as an intra-mitochondrial vehicle for the acyl group, which in the form of acyl-CoA acts as a substrate for the oxidation process, producing energy for sperm respiration and motility [7, 8]. It has also been reported to ameliorate the effects of reactive oxygen species (ROS) and free radical-induced oxidative stress (OS) and minimizing pathological disorders of sperm, like adenosine triphosphate (ATP) depletion leading to insufficient axonemal phosphorylation, lipid peroxidation as well as loss of motility and viability [9, 10]. As LC has been reported to act as a potent antioxidant with very less side-effects, researchers are now considering its implementation as a treatment for female infertility [5, 11, 12]. It is well known that OS also affects female reproduction in various ways, including lipid peroxidation of oocytes [13], fertilization and embryo development in animals [14].

Similarly, acetyl-L-carnitine (ALC), the primary acetyl ester of LC has been reported to have beneficial impacts on reproductive functions through its antioxidative effects [7, 8, 15]. ALC plays an essential role in intermediary metabolism, acting as a donor of acetyl groups and facilitating the transfer of fatty acids from cytosol to mitochondria during beta-oxidation in animals [15]. ALC has also been reported to exert cholinomimetic effects and to modulate the gamma-amino butyric acid (GABA) system. Since it is abundant in hypothalamus, it can affect the neuronal activity and thus hypothalamo-pituitary gonadal (HPG) axis to exert its impact on female reproduction [16].

To date, many research studies have already been carried out in human [11, 12, 17, 18] and animal models [16, 19–21] using carnitines to treat female infertility as well as to improve reproductive performance. It has also been used in ART in an attempt to solve similar problems [22, 23].

This review summarizes the effects of both LC and acetyl-L-carnitine (ALC) on female fertility with detailed scrutiny of the reports published till date. It provides a glimpse of human, animal and in vitro experimental studies carried out to solve the problems of female infertility. It also provides its possible mechanism of actions when supplemented individually or in combination with other nutrients.

### L-carnitine and acetyl L-carnitine: Physiological actions

LC is a small water-soluble molecule that plays an enormous role in normal physiology. It was first isolated from bovine muscle in 1905 by two Russian scientists [24], and only the L-isomer was found to be bioactive [4]. In mammals, ingested LC is absorbed from the small intestine via active transport and passive diffusion [25]. LC is then incorporated into the total body carnitine pool that includes uncharged LC, and short chain carnitine esters (acylcarnitines) such as ALC and propionyl L-carnitine (PLC). LC is also synthesized in the liver and kidneys via methylation of L-lysine [26]. A strict vegetarian diet may lead to deficiency of LC since red meat is the richest source of lysine. The accumulation of LC occurs in liver, skeletal muscle, heart, brain and testis [27]. The body tissue distribution studies show a very high concentration of LC in mature spermatozoa and luminal fluid of cauda epididymis [26]. Under physiological conditions, the carnitine pool (LC and ALC) remains in equilibrium because of the actions of local transferases and tubular reabsorption in the kidney [28].

**Fig. 1:**
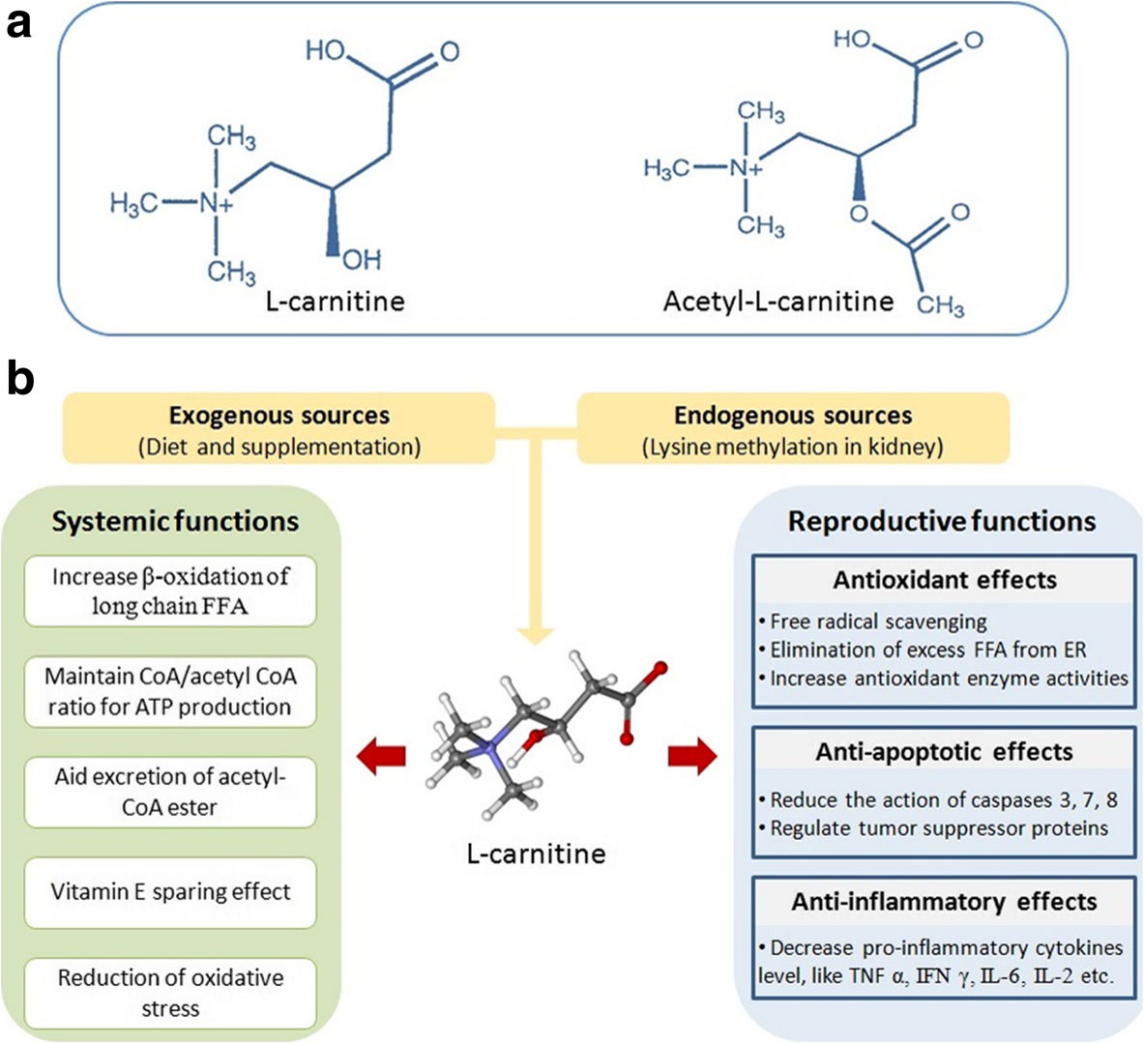
(**a**) Molecular structures of L-carnitine and acetyl-L-carnitine, (**b**) systemic and reproductive functions of L-carnitine. CoA, coenzyme A; ER, endoplasmic reticulum; FFA, free fatty acid; IFN, interferon; IL, interleukin; TNF, tumor necrosis factor

Carnitines belong to one of the special classes of nutrients called ‘quasi-vitamins’ or ‘conditionally-essential’ nutrients [29]. LC intake is widely regarded as one of the most effective ways to promote endurance, burn fat, and shorten post-workout recovery [27]. In peripheral tissues, it helps in β-oxidation by transporting medium and long chain fatty acids into the mitochondria [30]. It is also important in maintaining cell membrane stability through its involvement in acetylation of membrane phospholipids and amphiphilic actions [31, 32]. LC also prevents DNA damage induced by the detrimental actions of free radicals [33]. It also functions as a stabilizer of a low acetyl CoA/CoA concentration ratio and as an acetyl store to supply energy in carbohydrate and lipid metabolic pathways [34] (Fig. 1).

### L-carnitine and acetyl L-carnitine in female fertility

Several research studies have been carried out till date in human patients, animal models and in ART settings to investigate the effectiveness of LC and ALC in the improvement of female fertility [5, 22].

#### L-carnitine or acetyl L-carnitine - which carnitine is suitable for the treatment of female infertility?

Though a minute functional difference between LC and ALC has been reported, both are used in reproductive biology research to improve mitochondrial functions to treat infertility [5, 8, 9]. ALC is used most often associated with the improvement of antioxidant/anti-aging effects, while LC is commonly used to improve the body’s ability to oxidize fat cells which assists in the production of energy and burning fat [27]. During oocyte development, it has been reported that the cumulus-oocyte complex (COC) plays an essential role in lipid metabolism and energy production. Thus, in oocytes, maintenance of proper lipid oxidation with/without a minimum production of free radicals is crucial to preserve its quality [30]. Though it is well documented that both forms of carnitine possess antioxidant properties [10], it has been suggested in some reports that ALC is more effective in combating ROS-induced oxidative damage compared to LC [15]. Thus, studies focusing on antioxidant defense-mediated improvement of female fertility preferentially utilize ALC, whereas, studies demonstrating improved lipid metabolism-induced enhancement of oocyte quality, and thereby fertility, tend to use LC. Other acylcarnitines are also used in reproductive biology research, but to a lesser extent [35]. In 2013, Varnagy et al. reported the profiling of several acylcarnitines in the serum and follicular fluids of women undergoing IVF. They provided suggestive evidence that in IVF patients with better reproductive potential (higher number of oocytes and/or viable embryos), the LC/acylcarnitine pathway appears to be upregulated while the endogenous carnitine pool is diminished, which consequently improves oocyte quality [35]. In the following sections, we will provide evidence of LC and/or ALC supplementation and their role in female fertility.

#### Human studies

Most of the human experiments were carried out using LC as a supplement to alleviate/combat the problem of female infertility [11, 18, 36] (Table 1). Several studies found that both LC and ALC supplementation improves disorders such as polycystic ovary syndrome [12], endometriosis [19] and amenorrhea [17]. Carnitines are reported to increase gonadotropins and sex hormone levels as well as improve oocyte health [17]. However, the effects of LC on endometriosis are still a matter of debate [19, 29, 37, 38]. The possible mechanistic pathways through which LC could exert its effects on endometriosis will be discussed in a subsequent section.

**Table 1:** Human and animal studies involving carnitine supplementation to improve female fertility/reproductive status

Study aim	Carnitine(s) supplementation (dose and duration)	Study design/Subjects	Outcomes relevant to reproduction	Reference
*Human Studies*				
Single center, prospective study to evaluate whether ALC modulates opiatergic pathway in a group of patients with stress-induced amenorrhea	1 g/day ALC orally for 16 weeks	24 patients (aged 21–32 years) with FHA for the last 6 months were subdivided into 2 groups: Group A (hypogonadotropic patients, plasma LH levels ≤3 mIU/ml, *n* = 16) Group B (normogonadotropic patients, plasma LH levels > 3 mIU/ml, *n* = 8)	• ALC administration significantly modulates GnRH and LH secretion in hypothalamic amenorrhea in hypogonadotropic patients • ALC probably affects neuroendocrine control of gonadotropin secretion by modulating opiatergic control of GnRH-LH secretion • Data support the use of ALC as a therapeutic drug for stress-induced reproductive disorders with low LH levels or hypogonadotropic condition	[17]
Single center, double blinded, randomized controlled clinical trial to evaluate the effectiveness of LC on improving the ovulation and pregnancy rates as well as adverse metabolic indices in clomiphene-resistant PCOS women	3 g/day LC orally from day three until day seven of the cycle	170 clomiphene resistant PCOS women (aged less than 35 years) were randomly allocated into 2 groups: Group A (*n* = 85) received 250 mg clomiphene citrate plus LC Group B (*n* = 85) received 250 mg clomiphene citrate with placebo	• The combination of LC and CC significantly improved both the ovulation and the pregnancy rates in clomiphene-resistant PCOS women • The number of stimulated follicles reaching ≥17 mm diameter was significantly higher in Group A as compared to Group B • Endometrium at the time of hCG administration was significantly thicker in Group A • Serum E2, on the day of hCG administration, was significantly higher in the LC group • Pregnancy occurred in 42/85 cycles in Group A (54.5%) and 5/85 cycles in Group B (5.8%) and the difference was statistically significant. • The miscarriage rate was lower in Group A (2/85; 2.3%) than in Group B (4/85; 4.7%) (*p* = 0.67)	[11]
Before-after clinical trial to examine the effect of adding LC to PCOS patients who were resistant to clomiphene citrate and gonadotropin	2 g LC orally every 12 h, given from the third day of treatment with clomiphene citrate and gonadotropin until the hCG injection	50 PCOS patients (aged 20–35 years) either received LC or did not receive LC	• LC-treated women experienced the growth of dominant follicles (64%, 32/50 therapeutic cycles) and displayed a positive pregnancy test (20%, 10/50 therapeutic cycles) • LC-supplemented women had increased left ovarian follicle size compared to those without LC, however right ovarian follicle size was not significantly different between both groups • LC supplementation increased the mean endometrial thickness compared to those without LC.	[18]
Prospective, randomized, double-blind, placebo-controlled trial to determine the effects of oral LC supplementation on weight loss, and glycemic and lipid profiles in women with PCOS	250 mg LC orally for 12 weeks	60 overweight PCOS (aged 18–40 years) were randomized to receive either LC (*n* = 30) or placebo (*n* = 30)	• LC supplementation reduced body weight, body mass index, waist circumference and hip circumference and improved glycemic control in PCOS patients compared with placebo. • LC supplementation did not affect lipid profiles or free testosterone levels compared with placebo	[12]
Oral LC supplementation on mental health and biomarkers of OS in women with PCOS	250 mg LC for 12 weeks	60 patients: randomized, double-blind, placebo-controlled study	• LC supplementation improved general and mental health parameters • LC supplementation improved TAC, MDA/TAC ratio and decreased lipid peroxidation	[40]
*Animal Studies*				
LC supplementation on endometriosis in BALB/c mice	25 mg/ml/d LC for 7 days	Young female BALB/c mice of 4–8 weeks of age	• Increased concentration of LC in serum • Decreased levels of IFN-γ, TNF-α, IL-6, IL-2	[19]
LC on reproductive hormones and organs of pregnant mice	0.5 and 1.0 mg/kg LC from day-1 until parturition	Female Swiss albino mice of 12–14 weeks of age	• Significantly increased FSH, LH and estradiol levels • Increased litter size, weight of reproductive organs and thickness of endometrium	[42]
ALC on HPG axis of female Sprague-Dawley rats	50 mg/kg/d ALC for 2 consecutive estrous cycles	Female Sprague-Dawley rats of 3 months of age	Improved hormonal secretions through HPG axis: increased GnRH, LH, estradiol and progesterone levels	[16]
LC on OS parameters in oophorectomized rats	100 mg/kg/d LC during post-castration day-21 to -35 (14 consecutive days)	Female adult Wistar rats	Decreased MDA, and NO levels, and improved TAC and OS index	[49]
Reproductive performance of sows on LC-supplementation	125 mg/d LC (pregnancy), 250 mg/d LC (lactation)	300 sows (Leicoma)	Improved litter size and piglets' body weight gain, decreased number of stillborn piglets	[51]
LC in gestating sow diet on fetal development	50 ppm in diet for 3 reproductive cycles, until day-110 of gestation	232 gestating sows	Increased leanness and muscle density in offspring	[52]
Dietary LC on laying hen performance and egg quality	500 mg/kg LC diet for 84 days	180 Isabrown laying hens of 27-weeks of age	Improved egg quality parameters including albumin content	[50]
LC on reproductive traits of white leghorns	125 ppm LC until 37-week of age	720 White leghorns	Improved total lipid and LC content in yolk	[43]
LC in cows during transition and high lactation period	2 g/d LC for 5 weeks	262 dairy cows (German Holstein)	Improved metabolic status during lactation period, increased milk protein content	[53]

*ALC*, Acetyl-L-carnitine, *FHA*, Functional hypothalamic amenorrhea, *HPG* Hypothalamo-pituitary-gonadal axis, *LC* L-Carnitine, *MDA* Malondialdehyde, *NO* Nitric oxide, *OS* Oxidative stress, *PCOS* Polycystic ovary syndrome, *SCC* Somatic cell count, *TAC* Total antioxidant capacity

#### Carnitine and PCOS

Polycystic ovary syndrome (PCOS) is a common female reproductive endocrinopathy. The main pathophysiological mechanisms underlying this syndrome include obesity, insulin resistance and hyperinsulinemia. Samimi and colleagues found that LC supplementation (250 mg per day orally for 12 weeks) lead to significant reduction in body weight, BMI, waist and hip circumference respectively as well as improved glycemic control in women with PCOS (mean age 24.8 ± 5.5 years) [12]. This study indicated that LC supplementation improves PCOS by decreasing blood glucose levels and opposing insulin resistance [12], which could perhaps be attributed to LC-induced increase in beta-oxidation of fatty acids and basal metabolic rates [39].

Women with PCOS also have an imbalance between male and female hormones as their ovaries tend to produce androgens in excessive amounts. One study suggested that hyperandrogenism and/or insulin resistance in the non-obese women with PCOS may be associated with decreased total serum LC levels [36]. Fenkci’s group had measured the serum total LC levels in non-obese women with PCOS (*n* = 27; aged between 16 to 37 years) in comparison to that of healthy adult women (*n* = 30). They demonstrated these PCOS patients have significantly lower total LC (40.5 ± 5.7 μmol/L; in control: 91.1 ± 15.2 μmol/L), but higher levels of dehydroepiandrosterone (DHEA), testosterone, luteinizing hormone (LH), low-density lipoproteins (LDL) and fasting insulin compared to healthy women [36].

Another common feature of PCOS is chronic anovulation. The standard approach to treat women with anovulatory infertility, secondary to PCOS, is to administer clomiphene citrate to induce ovulation. However, some women do not ovulate despite receiving increasing doses of clomiphene citrate, and are therefore considered to be clomiphene citrate-resistant. In a single center, double blinded, randomized controlled clinical trial, Ismail et al. studied the effect of LC supplementation on clomiphene-resistant women with PCOS. All subjects received 250 mg clomiphene citrate daily from day-3 until day-7 of the cycle. In addition, women in the treatment arm (*n* = 85) were supplemented daily with 3 g of LC orally (day-3 until day of first positive pregnancy test), while those in the control arm (n = 85) received a placebo. Follicular maturation was monitored using transvaginal ultrasonography on all patients on day-7 and day-9, and subsequently adjusted according to individual response [11].

Ismail’s group found that LC supplementation along with clomiphene citrate treatment improved both ovulation (64.4% vs.17.4%; < 0.0001) and pregnancy (51.5% vs. 5.8%; *p* < 0.0001) rates in clomiphene-resistant women with PCOS. LC supplementation seemed to improve the number and rate at which the stimulated follicles developed (to a diameter of ≥17 mm for induction of ovulation), increase beta-oxidation and oocyte maturation as well as increase serum levels of both estradiol (E_2_) and progesterone. LC supplementation not only improved reproductive health, but also enhanced the patients’ lipid profile and body mass index (BMI) [11].

A typical alternative treatment to induce ovulation in clomiphene citrate-resistant PCOS patients is gonadotropin therapy. However, some women with PCOS fail to respond to both these treatments. Latifian et al. studied the effects of LC on 50 infertile women with PCOS who were both clomiphene- and gonadotropin-resistant. These women (mean age 27.98 ± 4.61 years) showed improvement following LC supplementation, which was given 2 g orally every 12 h (from the third day of the ovarian stimulation cycle onwards until the HCG injection). The LC-treated women in this study experienced the growth of dominant follicles (64%, *n* = 32) and displayed a positive pregnancy test (20%, *n* = 10). Moreover, LC was observed to increase the mean endometrium thickness as well as to inculcate positive changes in the left ovarian follicle size [18].

Not only does LC supplementation improve reproductive parameters in PCOS patients, but it can also confer positive effects on other health parameters in these women. This was demonstrated in a recent study whereby oral LC supplementation (250 mg for 12 weeks) among patients with PCOS improved total antioxidant capacity (TAC), decreased lipid peroxidation and enhanced general and mental health parameters [40].

#### Carnitine and functional hypothalamic amenorrhea

Functional hypothalamic amenorrhea (FHA) is a form of hypogonadotropic hypogonadism that results from an aberration in the pulsatile release of hypothalamic gonadotropin-releasing hormone (GnRH), which causes decreased gonadotropin release leading to reduced estradiol production in the ovary [41]. ALC administration (1 g/day orally for 16 weeks) in patients suffering from FHA (*n* = 24, age range 21 to 32 years) was found to increase LH levels by counteracting certain neuroendocrine pathways that have an inhibitory effect on the reproductive axis [17]. Genazzani and co-workers proposed that ALC acts through the opioidergic pathway and alters protein/hormone functions by acetylating -OH groups in amino acids like serine, threonine or tyrosine, thereby improving their functions. They concluded that ALC has positive therapeutic effects on the reproductive axis in hypogonadotropic women with FHA and improves the condition by acting through the HPG axis [17].

#### Animal studies

Numerous research studies have been carried out in animal models (murine and farm animals) to investigate how LC and ALC improve female reproductive functions [16, 19, 21, 42–44] (Table 1). Carnitines have been showed to have beneficial impacts in most of the studies involving PCOS, endocrine disorders and other cases of infertility. However, as alluded earlier, carnitines appear to possibly exert mixed effects on endometriosis [19, 29, 37, 38].

- Mice and rat models

*Carnitine, cytokines and immune response*

Despite the aforementioned beneficial effects of LC and ALC treatment on female fertility, Dionyssopoulou et al. found that carnitine supplementation modified the production of various cytokines, such as interferon-γ, tumor necrosis factor-α, interleukins-2, − 4, − 6, vascular endothelial growth factor, granulocyte-macrophage colony stimulating factor, and insulin-like growth factor-1 in serum and peritoneal fluid as tested using the immunofluorescence or ELISA technique [19]. Furthermore, carnitine treatment altered the percentage of immune cells (macrophages, T cells, CD4^+^ and CD8^+^ cells) in peritoneal exudates and uterine cells. Immune modifications such as these as well as the presence of cytokines and growth factors in the peritoneal cavity may contribute to the development of endometriosis. The potential role of prostaglandins (PG), including that of PGE_1_ and PGE_2_, are directly associated with inflammation and could affect the function of immune cells. It was found that while inhibition of PGE_1_ further increased the levels of certain cytokines in serum and peritoneal fluid [45], inhibition of PGE_2_ reversed these values toward that of control [46]. Thus in presence of LC, it was suggested that PGE_2_ plays a role in the mechanistic pathway leading to endometriosis, while PGE_1_ may potentially be involved in suppressing the production of immune cells and inflammatory response [29].

*Carnitine and the HPG axis*

In 1992, Krsmanovic et al. examined the impact of ALC (50 mg/kg/day for a month) on the HPG axis in female rats and found that serum LH and prolactin levels were increased during the proestrous and estrous phases, while serum E_2_ levels were increased during the estrous phase of the cycle. They have also noted an increase in uterine weight during both the proestrous and estrous phases of ALC-treated animals. Release of basal GnRH was found to be increased during the proestrous and diestrous I in hypothalamic slices of ALC-treated rats, which could mean that ALC stimulates the secretory activity of GnRH-producing hypothalamic neurons. In contrast, no alteration in pituitary functions was detected following ALC treatment in vitro. From these results, it was concluded that ALC may act through the HPG axis and cause gonadotropin release and this action is dependent on the cycle stage [16].

*Carnitine and oocyte quality*

Virmani et al. reported in 2015 that treatment of mice with both LC (0.4 mg/mouse) and ALC (0.12 mg/mouse) resulted in increased number of mature oocytes and less degradation of oocytes. They concluded that LC and ALC co-treatment showed better response in maintaining the quality and quantity of oocytes in mice. In females, an age-related decline in the number and quality of follicles and oocytes have already been documented [47]. Aging and ROS affects many biochemical pathways of developing oocytes that may produce a deleterious impact on oocyte health and in this aspect, LC supplementation has showed some beneficial roles. Christiana’s group investigated another potential effect of LC through their study in which young BALB/c mice were given LC supplementation (2.5 mg/day/mouse for 7 days), superovulated and then mated with healthy males. They showed that 7 day-LC administration alters the lipid body content in pre-implantation embryos and did not result in live births in these females. This is because lipid body content is an important contributory factor towards healthy oocyte and embryo development and therefore its alteration may lead to infertility [37]. Conversely, a recent report by Virmani et al. (2017) showed that supplementation of carnitines (LC 0.4 mg/mouse + ALC 0.12 mg/mouse) to 8-week-old female CD1 mice not only improved oocyte formation but also increased the number of live birth in superovulated mice [48].

*Carnitine and pregnancy-related parameters*

Fakhrildin and Flayyih reported an improvement in weight of the reproductive organs (ovaries, uterine horns, vagina) and endometrial thickness, litter sizes as well as serum LH, FSH and E2 levels in pregnant mice (Swiss albino strain females aged between 12 to 14 weeks) given 0.5 and 1.0 mg/kg LC compared to the control group. As the parameters measured did not differ significantly between the two LC doses used, the study concluded that a low dose of LC was sufficient to render positive effects on pregnancy and offspring outcomes in pregnant mice [42].

*Carnitine and oxidative stress*

Canbolat and co-worker’s study investigated the antioxidant property of LC (100 and 500 mg/kg/day) on oxidative stress parameters (nitric oxide (NO), malondialdehyde, total antioxidant status, total oxidative stress (OS), and OS index in the kidney, liver and heart, as well as sera of bilateral oophorectomized rats. They have reported LC exhibits antioxidant effects in different tissues with less NO production, lipid peroxidation and OS index when supplemented with 500 mg/kg/day LC intraperitoneally for 14 consecutive days in surgically menopausal rats [49].

b) Farm animal models

Several studies have also looked into the effects of carnitine supplementation on improving the rate of ovulation and fertilization [20], egg quality [44, 50], number of litters born [20, 51] etc., of farm animals such as pigs, chickens and cows.

*Carnitine and ovulation and fertilization rates*

Samland et al. examined the effect of dietary supplementation of LC (200 ppm) on the ovulation and fertilization rate of gilts (young female adult breeding pigs that have not yet farrowed). After two weeks, the gilts fed with added carnitine were found to have increased rate of ovulation, but decreased fertilization rate of the recovered embryos. However, the report did not offer an explanation as to the possible cause of decreased fertilization rates despite increased ovulation rates in this group of gilts [20].

*Carnitine, embryo development and post-natal growth*

Zhai and co-workers examined the effect of dietary supplementation of LC (125 ppm) on the reproductive traits of male and female White Leghorn chicken. They reported that the yolk of fresh eggs retrieved from LC-hens that were inseminated with semen from roosters consuming either the control or the LC-diet contained higher concentrations of LC but decreased hatchling yolk sac weights and yolk sac lipid content compared to that of control hens. This suggests that LC encourages fat utilization in the developing embryos [43].

In another study using gestating sows (mature female pigs that have farrowed at least once before the current gestation), Musser et al. demonstrated that a 50 ppm LC supplementation in gestation sow diet improved muscle fiber development by increasing the leanness and muscling of the fetal piglet. These changes could perhaps improve postnatal growth in the piglets [52].

*Carnitine and egg quality*

In another study on the performance and egg quality parameters of Brown egg-type laying hens, Corduk and Sarica reported that supplementation of 500 mg LC per kg of low energy diets containing either sunflower or palm oil increases the eggshell breaking strength. Moreover, the addition of LC into normal diet containing palm oil increased the albumen index (calculated from the albumen height, length and weight) of the eggs [50]. Greater eggshell breaking strength and a higher albumen index indicates higher egg quality.

*Carnitine and milk production and quality*

Pirestani and colleagues evaluated the effect of LC supplementation (50 g/day/cow) for 40 days on milk somatic cell count (SCC) (as a measure of the milk quality) and several reproductive indices in Holstein dairy cattle. They reported a significant reduction in milk SCC (indicating less mastitis or enhanced udder immunity and therefore better quality of milk) and improvement in the reproductive indices. In addition, the study found that a combination of LC and a vitamin-like essential macronutrient, choline (60 g/day/cow) further reduced milk SCC and improved the reproductive indices [21].

In another study on German Holstein dairy cows, Scholz’s group showed that supplementation with 2 g of LC/cow (contained in 10 g of a rumen-protected product) for three months improved metabolic health during the critical periods of transition and high lactation and showed a trend of improving milk production during the initial 2-months after calving. Furthermore, LC-supplemented cows had a lower insemination index and increased conception rate compared to controls [53].

#### In vitro studies and assisted reproduction

Following reports on the antioxidant properties of LC and ALC and its beneficial effects on female fertility, carnitines have been used in in vitro studies focusing on the improvement of oocyte health and maturation, embryo development as well as in assisted reproduction [54, 55] (Table 2). In the last two decades, a good number of studies have been published that showcase the impact of carnitine supplementation on female fertility. Of late, it has been used to minimize ROS-mediated delayed embryonic development in culture medium, high DNA fragmentation and development of morphologically abnormal blastocysts after prolonged culture [6, 23, 56].

**Table 2:** In vitro studies with carnitine supplementation to improve oocyte quality, maturation and embryo development

Study aim	Carnitine dose	Study design/Subjects	Outcomes	Reference
LC on antagonizing the harmful effect of TNF-α, apoptosis, and oxidative stress on mouse embryo development.	LC was dissolved in HTF culture medium in concentrations of 0.3 and 0.6 mg/mL	• 500 mouse embryos were divided into three groups and incubated with either AD 0.005 mg/mL, H_2_O_2_ 500 mmol/L, or TNF-α 500 ng with and without LC 0.3 or 0.6 mg/mL • For anti-apoptotic effect: All groups were incubated at 37C in 5%CO_2_ for 4 h and transferred to HTF medium and incubated until 48 h for formation of the blastocyst stage. • For anti-oxidant and anti-proliferative effect on TNF-α: All groups were incubated at 37C in 5% CO_2_ for 72 h until the formation of blastocyst stage. • Embryo staining by TUNEL was done to detect blastomere DNA damage	• Significant improvement in percentage BDR was seen at LC 0.3 mg/mL compared with the control (*p* < 0.006) • L-Carnitine at 0.3 and 0.6 mg/mL significantly reduced the blocking effect of AD, H_2_O_2_, and TNF-α and significantly decreased the level of DNA damage	[6]
LC on oocyte cytoskeleton and apoptosis in peritoneal fluid from patients with endometriosis	0.6 mg/mL of LC	• Peritoneal fluid was collected from 23 women suffering from endometriosis and 15 patients with tubal ligation who underwent laparoscopy • LC was diluted 1:1 with peritoneal fluid and cryopreserved mouse embryos were matured in that medium	Significantly improved microtubule and chromosome structure and decreased embryo apoptosis	[38]
LC on oocyte maturation and parthenogenetic embryos in pigs	0.25, 0.5, 1.0 and 2.0 mg/mL of LC in IVM medium	• Porcine ovaries were collected from prepubertal gilts and matured in medium containing various concentrations of LC. • LC was added in IVC medium to examine developmental competence of parthenogenic embryos	LC addition during IVM improved developmental potential of oocytes, and also quality of parthenogenic embryos by improving nuclear maturation and preventing OS and apoptosis	[14]
LC on lipid metabolism and in vitro maturation of porcine oocyte	0.3 to 10 mg/mL of LC	Ovaries from prepubertal cross-bred gilts were collected and IVF and IVC was performed in media containing LC	Enhanced mitochondrial functions, lipid metabolism for nuclear and cytoplasmic maturation of porcine oocytes	[54]
LC on oocyte maturation and embryo development	10 mM of LC in IVM medium	• Porcine ovaries were collected from 6 to 7 months old prepubertal gilts • Comparison of GSH, ROS levels and developmental gene expression in LC supplemented and non-supplemented group	• Reduced OS with increased GSH synthesis in LC supplemented group • Increased expression of developmental genes	[23]
LC on maturation rate of buffalo embryos	0.3, 0.6 and 1.2 mM/mL of LC	Oocytes were collected from Swamp buffalo and treated with various concentrations of LC	Significantly higher metaphase II oocytes than control group with faster maturation rate	[66]
LC on bovine embryo development and their cryotolerance	1.1518 mM and 3.030 mM of LC	• Oocytes were collected from bovine ovaries • IVF and IVC was performed in media containing LC	Improved cryotolerance, lipid metabolism in embryos	[68]
LC on vitrification of mouse germinal vesicle stage-oocytes and their in vitro maturation	3.72 mM (0.6 mg/mL) of LC in IVM medium	• B6.DBA cross-bed mice were superovulated and oocytes were collected • Oocytes grown in LC-supplemented IVM medium	Increased number of metaphase II oocytes and improved mitochondrial distribution in oocytes	[55]
ALC on lamb oocyte blastocyst rate, mitochondrial DNA copy number	2 mM of ALC in IVM medium	Prepubertal lamb oocytes were collected and matured in medium containing LC	Increased cytoplasmic volume of oocyte with more lipid droplets, but no alteration in mitochondrial volume, number or DNA copy number	[22]
LC on maturation of mouse embryos	0.3 and 0.6 mg/mL of LC	• Immature oocytes were collected from NMRI mice ovaries and treated with LC • Cleavage rate, BDR and GSH were evaluated	Improved implantation developmental competence and nuclear maturation of oocytes and increased GSH	[64]
LC on bovine blastocyst development	0.1, 0.5 and 1.0 mg/mL of LC in IVM medium	• Oocytes were collected from bovine ovaries and matured in medium containing LC • These oocytes were then subjected to IVF with fresh semen • Number of embryos and total cell count was performed	Improved developmental potential: increased number of oocytes and embryos with higher total cell count	[70]
LC in OS and antioxidant profile in sheep embryos produces in vitro	2.5, 5, 7.5 and 10 mM of LC in maturation medium	Oocytes were collected from sheep ovaries and matured in medium containing LC. These oocytes were then subjected to IVF with fresh semen.	• Oocyte maturation, embryo development increased • LC reduced OS and ROS production, increased antioxidant enzyme activities	[56]
LC on in vitro maturation of ZP and development of mouse embryos	0.5, 1, 2 and 4 mg/ml of LC	• Mice were superovulated and mating was carried out with males • 2-cell embryos were flushed from oviduct and cultured in LC containing medium • BDR, ZP thickness were measured	Increased number of blastocyst cells, ZP thickness and improved antioxidant activity	[71]

*AD* Actinomycin-D, *ALC* Acetyl-L-carnitine, *BDR* Blastocyst development rate, *GSH* Glutathione, *HTF* Human tubular fluid, *H*_*2*_*O*_*2*_ Hydrogen peroxide, *IVC* in vitro embryo culture, *IVF* in vitro fertilization, *IVM* in vitro maturation, *LC* L-Carnitine, *OS* Oxidative stress, *ROS* Reactive oxygen species, TNF-tumor necrosis factor, *ZP* Zona pellucida

During in vitro fertilization (IVF) procedures, embryo fragmentation due to apoptosis is a common occurrence that is well documented. Nonetheless, supplementation of culture medium with LC may confer protection to the developing immature cells. Pillich et al. have shown that ALC supplementation (0.3, 0.6 and 1.2 mM for 5 h and 24 h) in mouse fibroblast culture media stabilizes the mitochondrial membrane, increases energy supply to the organelle and protects the developing cell from apoptosis through the mitochondrial pathway [57]. In another laboratory, Abdelrazik and colleagues looked into the optimal dose of LC that is required for blastocyst development of mouse embryos. They have showed that 0.3 and 0.6 mg/mL of LC possesses anti-apoptotic effects as well as increased the rate of blastocyst development [33].

A marked increase in TNF-α concentration in granulosa cell cultures of women with endometriosis has been demonstrated [58–61]. Studies have also shown that increased levels of TNF-α restrict inner cell mass and trophectoderm proliferation in mouse blastocyst. At a concentration of 50 ng/mL, TNF-α was found to affect protein synthesis in mouse embryos in both morula and blastocyst stages [62, 63]. However, LC at the doses of 0.3 and 0.6 mg/mL were able to neutralize the anti-proliferative effects on TNF-α. LC supplementation in embryo culture medium also decreased DNA damage during development [6].

These observations were reaffirmed by Zare and co-workers who used the same doses (0.3 and 0.6 mg/mL) of LC supplementation during in vitro maturation of immature BCB+ (Brilliant Cresyl Blue positive) oocytes. LC-treated oocytes demonstrated an improved pre-implantation developmental competence (quality) after IVF, which is probably due to LC-induced improvement in the cytoplasmic and nuclear maturation of immature oocytes. LC at the doses used also exhibited an antioxidative effect during embryo development by reducing ROS levels in the maturation medium [64].

In another study, Mansour et al. used the same dose (0.6 mg/mL) of LC to demonstrate the protective effects of LC on oocytes and embryos against the toxic effects of peritoneal fluid in women with endometriosis. They have showed that peritoneal fluid of patients with endometriosis which was supplemented with LC had decreased apoptosis levels in the embryos and improved oocyte microtubular and chromosomal structure [38]. Bareh has also reported that LC-induced improvements in cytoskeleton could lead to reduced aneuploidy rates in an animal model [65].

In 2013, Phongmitr et al. added LC (0.3, 0.6 and 1.2 mg/mL) to culture media containing swamp buffalo oocytes and noted an improved nuclear maturation rate with a maximum number of metaphase II (MII) oocytes in the 0.3 mg/mL LC-supplemented group [66]. Moawad’s group later showed that LC supplementation (0.6 mg/ml or 3.72 mM) during vitrification/warming and in vitro maturation of germinal vesicle stage-oocytes increased the proportions of oocytes with normal MII spindles. They also reported that LC supplementation causes increased oxidative activity of mitochondria in the resultant MII oocytes without increasing the ATP levels in MII oocytes [55].

Ghanem and co-workers reported similar observations of increased antioxidant activity and improved blastocyst quality by way of higher embryo development rate and fewer number of apoptotic cells after supplementing LC (1.5 mM, equivalent to 0.3 mg/mL) in culture media of bovine blastocyst during the pre-implantation stage [67]. Similar studies conducted earlier have also shown higher counts of total cells [68, 69] and lower apoptotic cells [69] in bovine embryos cultured in 0.3 mg/mL L-carnitine.

Manzano’s group investigated the role of L-carnitine supplementation in the maturation of bovine oocytes and pre-implantation development of embryos. They reported improved developmental potential up to the blastocyst stage with higher blastocyst formation rate and increased total cell count and activity when the oocytes were matured in culture media added with 0.1–0.5 mg/mL LC. In a subsequent experiment, supplementation of the resulting zygotes with the same dose of LC in modified synthetic oviductal fluid medium displayed a higher blastocyst total cell count, but similar blastocyst formation rates as that of control [70].

Khanmohammadi and colleagues evaluated the effect of LC (0.5 mg/mL) supplementation on several indicators of embryo development and blastocyst quality such as thickness of zona pellucida, hatching rate and number of blastocyst cells. At a concentration of 0.5 mg/mL, LC exerted antioxidant properties, along with an enhancement of mitochondrial lipid metabolism to increase blastocyst cell number, blastocyst expansion and zona pellucida thinning, all of which promotes blastocyst quality leading to successful hatching and implantation. Conversely, LC at high concentrations (4 mg/ml) had a toxic effect on in vitro embryo development and blastocyst quality [71].

### Possible mechanisms of action of LC

#### Direct effects

LC has been reported to maintain cellular energy [34], reduce oxidative stress [72] and minimize cell death by apoptosis [33], which are necessary for proper oocyte growth and maturation of blastocyst. As discussed earlier, the cumulus-oocyte complex (COC) and its lipid metabolism are one of the prime regulators of oocyte maturation [73]. LC helps in the metabolism of COC lipids by transferring fatty acids into the mitochondria and by facilitating β-oxidation [30]. LC is taken up by the tissues via the electrogenic force of the voltage-gated Na^+^-channels. It uses the Na^+^-driven LC / organic cation transporter-2 (OCTN-2) for its transport into the oocytes [74].

**Fig. 2:**
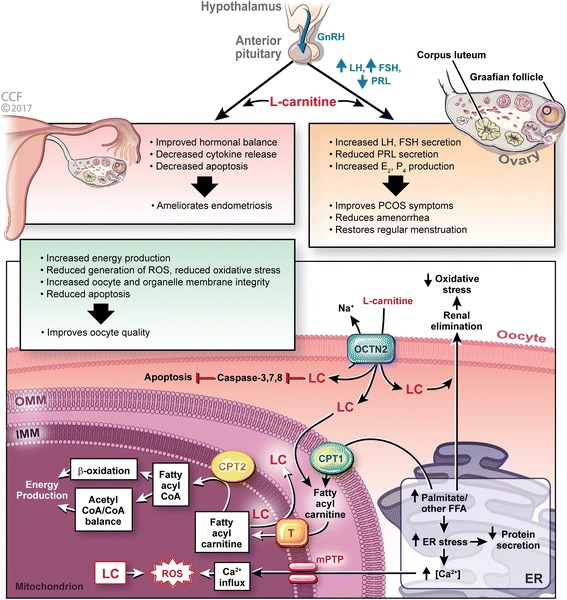
Mechanism of L-carnitine action on female fertility. LC enters the oocyte through OCTN2 and through its direct action on oocyte quality, it increases energy production by β-oxidation, eliminates excess palmitate from ER to reduce ER stress, scavenges free radicals to reduce oxidative damage and inhibits caspases to prevent apoptosis. It implies its indirect action through HPG axis by regulating the reproductive hormone levels and thus mitigates reproductive disorders such as PCOS and amenorrhea. In endometriosis, it improves hormonal balance, decreases the release of cytokines as well as apoptosis and thus ameliorates endometriosis. CPT1, carnitine palmitoyltransferase-1; CPT2, carnitine palmitoyltransferase-2; T, CoA, coenzyme-A; ER, endoplasmic reticulum; FFA, free fatty acid; FSH, follicle stimulating hormone; GnRH, gonadotropin releasing hormone; IMM, inner mitochondrial membrane; LC, L-carnitine; LH, luteinizing hormone; mPTP, mitochondrial permeability transition pore; OCTN2, organic cation transporter-2; OM, oocyte membrane; OMM, outer mitochondrial membrane; PCOS, polycystic ovary syndrome; PRL, prolactin; ROS, reactive oxygen species; T, translocase

In the oocytes, LC gets converted to ALC by carnitine palmitoyltransferase-I (CPT-I) in the outer mitochondrial membrane and CPT-II helps in the regeneration of carnitine from acyl-carnitine after the translocation of long chain fatty acids into the mitochondrial matrix [31]. Within the oocyte, LC plays a significant role in ER, mitochondria as well as in the ooplasm. In the mitochondria, LC is converted to ALC, and balances the acetyl CoA/CoA ratio to maintain glucose metabolism through the TCA cycle, yielding a higher energy production [34]. LC helps in minimizing the concentration of pyruvate which prevents entry into the TCA cycle to curtail energy production (Fig. 2).

In the mitochondria, LC also scavenges ROS through its antioxidant property. It transports palmitate and other long chain fatty acids to the mitochondria to facilitate their utilization through β-oxidation [5]. In the ER, it decreases the concentration of palmitate by transferring it to the mitochondria or by eliminating it from which it may cause lipotoxicity of the oocytes through oxidative stress [34]. It is reported that higher ROS levels decrease maturation by affecting the COC, and also decreases embryo development and blastocyst fragmentation [75]. LC also promotes cellular proliferation and decreases apoptosis by inhibiting TNF-α and other anti-proliferative agents [33]. In endometriosis, LC has been claimed to possess dual effects through the uterine secretion of prostaglandins. LC treatment has been shown to increase the production of both PGE_1_ and PGE_2._ It was suggested that PGE_1_ confers protection against endometriosis by attenuating the secretion of TNF-α, IFN-γ and interleukins (IL-2, IL-4 and IL-6), while PGE_2_ induces cytokine release and thus endometriosis through its apoptotic and inflammatory functions [19, 46]. However, further robust studies are required to corroborate the findings regarding the role of LC on endometriosis.

Thus, in summary, free LC or ALC serves three important functions in oocytes through its direct action: first, it increases energy production by transferring palmitate into mitochondria and maintaining acetyl CoA/CoA ratio; secondly, it reduces oxidative stress and lipotoxicity by scavenging free radicals and removing excess palmitate from the ER, and finally it promotes oocyte growth and maturation by decreasing the rate of apoptosis.

#### Indirect effects

LC and ALC have also been reported to affect the HPG axis to promote reproductive hormone secretion [16, 17, 76]. It has been known that among the neural centers, LC’s concentration is highest is in the hypothalamus [77]. In the hypothalamus, LC has been reported to decrease neuronal cell death and damage associated with aging [78] by its cholinomimetic activity [79]. It is reported that LC increases GnRH secretion from the hypothalamus by acting on the HPG axis [16, 17] and causing K^+^-induced depolarization in hypothalamic neuronal cells to increase its secretory activity [80, 81]. They have shown that treatment with ALC increases serum levels of other reproductive hormones, like estradiol, progesterone, LH and decreases prolactin in the usual fashion with different phases of estrous in animal [16, 17]. Through its indirect endocrine effect, it prevents PCOS, amenorrhea and other problems related to the female reproductive cycle.

Costa and Stevenson (1984) with the administration of equine chorionic gonadotropin (eCG) and pregnant mare’s serum gonadotropin (PMSG) showed that these components increases ovarian concentration of free LC and ALC, which shows the interaction between the direct and indirect mechanisms through cellular and hormonal regulations [82].

### Combination effects with other supplements

LC or ALC has not only been supplemented individually, in many experiments researchers have supplemented it in combination with other nutrients [20, 21, 44, 83–88] (Table 3). The results of combination treatments i.e. LC and/or ALC with other nutrients are not very well documented in female fertility, but in the last two decades, scientists have started applying LC and/or ALC with other nutrients to improve fertility [84]. It has been previously reported that LC (150 mg/kg/day) while combined with ginger extract (100 mg/kg/day) improves hormonal levels as well as reproductive performance in male rats [83]. It is also known that obestatin and LC co-treatment improves fat metabolism and obesity-induced male infertility [86].

**Table 3:** *S*tudies using a combination of carnitine and other nutrient supplementation to improve reproductive parameters

Study aims	Dosage and duration	Study design/Subjects	Relevant study outcomes	Reference
Effects of LC and CrNic on ovulation and fertilization rates in gilts	200 ppm LC + 200 ppb CrNic for 2 weeks prior to expected estrus	105 gilts Positive controls were fed 11 lbs./day of complete diet (flushing) Treatment group was fed 4 lbs./day of diet with/without various treatments	Increased ovulation and fertilization rate	[20]
Effects of dietary supplementation of LC and ascorbic acid in productive, reproductive, physiological and immunological performances of Golden Montazah aged breeder hens	100 and 200 mg LC/kg diet + 1 g ascorbic acid for 4 weeks	180 Golden Montazah hens + 18 cocks of 50 weeks old were randomly chosen. Birds were divided into 6 groups (each of 30 hens + 3 cocks), during the entire experimental period (from 50 to 70 weeks of age).	All groups fed diets of LC levels of 100 and 200 mg /kg diet alone and with 1 g ascorbic acid / kg diet were improved and recorded the best values of fertility and hatchability percentages. This improvement was increased with increasing LC level compared with the hens fed 1 g ascorbic acid /kg diet alone and the control group at the end of the experimental period.	[87]
CoQ10 and LC co-treatment in ovulatory response in rabbits	10 mg/kg CoQ10 + 40 mg/kg LC for 21 days	New Zealand White female rabbits of 5.3 months old	Higher ovulate rate, number of follicles, corpus luteum and increased number of embryos	[85]
LC and vegetable oil supplementation in broiler breeder fertility	60 ppm (females), 500 ppm (males)	–	In 5th and 6th week, egg production is increased with more cholesterol in egg yolk	[44]
Dietary LC and choline chloride on reproductive indices in Holstein Dairy Cattle	50 g/day	From 1-week pre-calving to 4-weeks after parturition	Decreased SCC and improved other immune indices	[21]
Both LC and ALC supplementations on ovulation and oocyte quality in CD1 mice	0.4 mg LC and 0.12 mg ALC/mouse/day for 3 weeks	Female CD1 mice of 8 weeks old	Decreased percentage of oocyte degeneration with more developing oocytes	[47]
NAC and LC on prevention of oocyte damage in women with mild endometriosis	1.5 mM of NAC with 0.6 mg/mL LC in follicular fluid	In follicular fluid sample from infertile women with endometriosis aged less than 38 years who underwent ICSI and induced in bovine oocytes	LC and NAC combination prevented oocyte damage in mild endometriosis	[84]
Effect of oral antioxidant and LC combination on IVF-ICSI outcomes	Vitamin C (180 mg), vitamin E (30 mg), zinc (15 mg), selenium (50 mg), LC tartarate (400 mg), folic acid (200 μg) and CoQ_10_ (40 mg) for 2 to 5 months at a daily dose of 2 capsules	Semen was taken for IVF and ICSI	• Enhanced semen quality • Improved sperm DNA integrity, fertilization rate, pregnancy rate, implantation rate, embryo quality as well as blastocyst development rate	[88]
LC plus ALC on neuroendocrine control of hypothalamic functions in FHA	LC fumerate (863 mg), ALC (250 mg), vitamin-C (90 mg), N-acetyl cysteine (50 mg), vitamin E (30 mg), iron (7 mg), pantothenic acid (6 mg), zinc (5 mg), vitamin B6 (2 mg), copper (0.5 mg), β-carotene (4.8 mg), folic acid (200 μg), vitamin-D3 (5 μg), selenium (27.5 μg), vitamin B12 (2.5 μg)	27 patients (aged 26.5 ± 2 years) with FHA for the last 6 months were subdivided into 2 groups: Group A (hypogonadotropic patients, plasma LH levels ≤3 mIU/ml, *n* = 15) Group B (normogonadotropic patients, plasma LH levels > 3 mIU/ml, *n* = 12)	• Carnitine administration significantly increases LH secretion in patients • Carnitine decreases cortisol and plasma amylase levels	[89]

*ALC* Acetyl-L-carnitine, *CoQ10* Coenzyme-Q10, *CrNic* Chromium nicotinate, *FHA* Functional hypothalamic amenorrhea, *LC* L-Carnitine, *ICSI* Intracytoplasmic sperm injection, *IVF* in vitro fertilization, *NAC* N-Acetyl cysteine

The role of LC in combination with other nutrients on female fertility is also demonstrated by various reports. As discussed earlier, Samland et al. (1998) have recorded the effect of LC supplementation on fertilization rate of gilts; they have not only recorded the effect of individual supplementation of LC (200 ppm), they have also combined it with chromium niconitate (CrNic) (200 ppb), an insulin promoter which increases insulin uptake by the cells. They have compared the outcomes of both cases in ovulation and fertilization rate and noted that LC individually potentiates ovulation, but when LC was combined with CrNic, it showed no alteration. They concluded that perhaps LC does not interact with CrNic to cause improvement in reproductive parameters [20].

In 2006, Adabi et al. in contrast, reported the supplementation of LC (60 ppm for females and 500 ppm for males) with vegetable fat powder (1.5%) and high lysine and methionine (0.3%) has increased fertility and hatchability in broilers. They also reported about their interaction as well as their role in lipid metabolism [44]. In 2011, Pirestani et al. supplemented LC individually (50 g/day/cow), as discussed earlier, as well as with choline chloride (60 g/day/cow) on SCC and reproductive indices of Holstein dairy cattle. They have reported choline chloride and LC co-administration has beneficial effects on SCC and reproductive indices, and that is better than individual LC supplementation [21].

In 2011, Hassan et al. supplemented different doses of LC (100 and 200 mg/kg diet) with vitamin C (1 g/kg diet) for 3 weeks and noted the reproductive performance of Golden Montahza laying hens, and reported increased egg production rate (2.1 and 2.7%) and egg quality [87]. In 2014, Kacem et al. formulated a special combination of vitamin C (180 mg), vitamin E (30 mg), zinc (15 mg), selenium (50 mg), LC tartarate (400 mg), folic acid (200 μg) and coenzyme-Q10 (40 mg). They have supplemented this formulation to male participants of a clinical trial of this drug for 2 to 5 months at a daily dose of 2 capsules before their semen has taken for IVF and intra-cytoplasmic sperm injection (ICSI). They have noted that the formulation has not only enhanced semen quality, it also improved sperm DNA integrity, fertilization rate, biochemical and clinical pregnancy rate, implantation rate, embryo quality as well as blastocyst development rate [88].

Cavallini et al. (2012), in their study, reported that 22 out of 33 male patients with severe idiopathic oligoasthenoteratozoospermia (OAT) of infertile couples whose partners had undergone at least one ICSI cycle, showed reduced sperm aneuploidy levels (assessed using fluorescent in situ hybridization performed on chromosomes X, Y, 13, 15, 16, 17, 18, 21 and 22) and improved sperm morphology after 3 months of treatment with LC 2 × 1 g/day, ALC 2 × 500 mg/day and cinnoxicam (a lipophilic NSAID) 1 × 30 mg/every 4 days [89]. Men with OAT have been reported to have elevated prostaglandin levels. NSAIDs act by reversibly inhibiting cyclooxygenase, which then inhibits the generation of prostaglandins and thromboxanes. Cinnoxicam and carnitines appear to have complementary mechanistic pathways, which facilitate the suppression of excess prostaglandin production, which could lead to improvement in sperm quality. The men with reduced sperm aneuploidy and improved sperm morphology as a result of the combination therapy in this study had significantly higher biochemical pregnancies, clinical pregnancies and live births after the treatment, despite having similar numbers of oocytes fertilized and embryos transferred [89].

In 2015, Abdel-Khalek et al. reported that coenzyme Q10 (10 mg/kg) and LC (40 mg/kg) for 21 days improved ovulatory response in rabbits, as well as embryo recovery and embryo vitrification in in vitro study [85]. Lastly, Virmani et al. (2015) combined both LC (0.4 mg/mouse) and ALC (0.12 mg/mouse) with micronutrients like zinc (4 ng/mouse), copper (0.8 ng/mouse) and iron (7 ng/mouse) and treated mice for 3 weeks daily to check the ovulation rate and oocyte quality. They have noted when LC and ALC are given in combination, its actions were better. Similarly, even when carnitines were given in combination with micronutrients, it showed better improvement in oocyte quality and quantity. They have also noted decreased rate of oocyte degeneration as well as increased rate of ovulation while supplemented with microelements. When they have tried this combination for IVF, they observed a better fertilization rate, 2-cell embryo development as well as birth rate [47].

Recently Genazzani et al. (2017) have repeated their experiment of combined LC and ALC effects on female fertility with a formulation containing LC fumerate (863 mg), ALC (250 mg), vitamin C (90 mg), N-acetyl cysteine (50 mg), vitamin E (30 mg), iron (7 mg), pantothenic acid (6 mg), zinc (5 mg), vitamin B6 (2 mg), copper (0.5 mg), β-carotene (4.8 mg), folic acid (200 μg), vitamin D3 (5 μg), selenium (27.5 μg), vitamin B12 (2.5 μg). They have reported that this formulation improves the condition of FHA and increases LH. They have also been shown to decrease stress-related cortisol and amylase levels [90].

Most of these reports pointing towards better interactions of LC and/or ALC with other nutrients and antioxidants to enhance fertility rate in female, including vitamin C [87, 88], vitamin E [88], zinc [47, 88], selenium [88], folic acid [88], coenzyme-Q_10_ [47, 88], vegetable oil containing high lysine [44] and many others [21, 47, 88].

### Combination therapies involving other antioxidants in female fertility

The female reproductive system is vulnerable to oxidative damage and without antioxidant interventions, the copious chain of oxidative reactions would continue to damage the system [58, 91]. Apart from LC and ALC, as discussed earlier, in conserving and regulating female reproductive functions, there are several groups of antioxidants known to quench free radicals to keep it healthier. These include vitamins (C, E, and β-carotene), some metallo-enzymes including glutathione peroxidase (GPx, containing selenium), catalase (CAT, containing iron), superoxide dismutase (SOD, containing copper, manganese and zinc). Dietary intake of these antioxidant vitamins and minerals prejudice the coordinated functioning of the total antioxidant system [92].

Within the ovary, various antioxidant systems (consisting of CAT, carotenoids, vitamin E, and glutathione) work to keep ROS under strict regulation. SOD catalyzes the superoxide decomposition into H_2_O_2_ and oxygen, and it has its effects in the theca interna cells of antral follicles. The theca interna cells in turn protect oocyte during its maturation phase from getting damaged by excess ROS. One more antioxidant factor imperative for healthy development of the ovarian follicle is transferrin (iron-chelating glycoprotein) that impedes the generation of ROS [91]. The connection of OS with endometrial cyclical alterations is well accepted and the SOD levels are demonstrated to rise in response to such oxidative changes during the end of the secretory phase, just prior to menstruation [93].

Vitamin C, one of the most commonly used antioxidant, is found in the cytosol of oocyte and extracellular fluid. Its supplementation to infertile women is in practice to treat luteal phase defects and recurrent abortions [58]. It is a versatile vitamin with antioxidant as well as collagen-stimulating properties. Studies revealed that when vitamin C is ingested through diet, the mature ovarian follicles actively take it up leading to its sequestration in the follicles rather than in the serum [58].

During in vitro fertilization (IVF) embryo transfer, vitamin C supplementation is provided to the patients during hormonal stimulation to ensure higher concentration of vitamin C in the follicular fluid [94]. It effectively breaks the chain of oxidative reactions to stop the promulgation of the peroxidative damage to the oocyte [91]. It is also claimed to elevate the chance of ovulation and protect oocyte from DNA alterations owing to oxidative damage. It helps to regenerate Vitamin E and glutathione. As discussed earlier, Hassan et al. supplemented vitamin C with LC and found improved reproductive performance in hens. This combination has been shown to increase egg production and egg quality thus attributing to reduced OS and improved lipid metabolism following LC-vitamin C co-treatment. Vitamin E (α-tocopherol) is found in the cell membrane of the oocyte and is evidently the first line of defense recruited to disrupt the chain of fatty acid peroxidation protecting the oocyte from further oxidative damage [92]. The follicular and tubular fluid contains taurine, hypotaurine and transferrin which protects the embryo from OS [95]. Glutathione has a role in the improvement of zygote development after the morula or blastocyst gains 2-cell blocks and it is also present in the oocyte as well as in the tubal fluid [96].

Hence, the interplay of antioxidants and their combined effort to combat free radicals, are constantly protecting the delicate organs belonging to the female reproductive system. Antioxidants take charge of their own regeneration as lipoic acid aids in the regeneration of Vitamin C, glutathione, vitamin E as well as of itself, glutathione recycles vitamin C which in turn recycles vitamin E that is also regenerated via CoQ_10_. This battalion of antioxidants, individually or in combination, if associated with LC or ALC, may lead to excellent results in improving female fertility. This can be suggested from the fact that the free radical quenching properties of the above-mentioned antioxidants as well as that of carnitines, when combined with the additional features of the carnitines, such as aiding fatty acid metabolism and energy production, would make the reproductive organs of female more robust.

## Conclusions

This review has summarized the information procured from various research works about the implications of LC and/or ALC upon female fertility. These carnitines, either individually or in combination with other nutrients and antioxidants, are potent to improve and/or restore female reproductive functions. This review has also proposed a mechanism of LC- and ALC-induced enhancement of female fertility, (a) directly by increasing energy production in oocytes and effectively quenching free radicals to provide protection against oxidative damage to the reproductive cells, and also (b) indirectly, by imposing their beneficial effects through HPG axis to ameliorate serum levels of hormones. Thus, considering the substantial qualities of these carnitines in female reproduction, they can be used both as reproductive biomedicines to treat female infertility as well as fertility boosters to improve the reproductive performance in humans and animals.

## Abbreviations

ALC: Acetyl L-Carnitine
ART: Assisted reproductive technology
BCB: Brilliant cresyl blue
CoA: Coenzyme-A
COC: Cumulus-oocyte complex
CPT: Cartinitine palmitoyltransferase
DHEA: Dehydroepiandrosterone
eCG: Equine chorionic gonadotropin
ER: Endoplasmic reticulum
FHA: Functional hypothalamic amenorrhea
FISH: Fluorescent in situ hybridization
GABA: γ-amino butyric acid
GnRH: Gonadotropin releasing hormone
HPG: Hypothalamo-pituitary gonadal axis
IFN: Interferon
IVF: In vitro fertilization
LC: L-carnitine
LDL: Low density lipoprotein
LH: Luteinizing hormone
MII: Metaphase-II
NO: Nitric oxide
OCTN: Organic cation transporter
OS: Oxidative stress
PCOS: Polycystic ovarian syndrome
PG: Prostaglandin
PLC: Propionyl L-carnitine
PMSG: Pregnant mare’s serum gonadotropin
ROS: Reactive oxygen species
SCC: Somatic cell count
TNF: Tumor necrosis factor
